# Content characteristics predict the putative authenticity of COVID-19 rumors

**DOI:** 10.3389/fpubh.2022.920103

**Published:** 2022-08-10

**Authors:** Jingyi Zhao, Cun Fu, Xin Kang

**Affiliations:** ^1^College of International Studies, Southwest University, Chongqing, China; ^2^School of Foreign Languages and Cultures, Chongqing University, Chongqing, China; ^3^Research Center for Language, Cognition and Language Application, Chongqing University, Chongqing, China

**Keywords:** logistic regression model, content characteristics, authenticity, COVID-19 rumors, linguistic

## Abstract

Rumors regarding COVID-19 have been prevalent on the Internet and affect the control of the COVID-19 pandemic. Using 1,296 COVID-19 rumors collected from an online platform (piyao.org.cn) in China, we found measurable differences in the content characteristics between true and false rumors. We revealed that the length of a rumor's headline is negatively related to the probability of a rumor being true [odds ratio (*OR*) = 0.37, *95% CI* (0.30, 0.44)]. In contrast, the length of a rumor's statement is positively related to this probability [*OR* = 1.11, *95% CI* (1.09, 1.13)]. In addition, we found that a rumor is more likely to be true if it contains concrete places [*OR* = 20.83, *95% CI* (9.60, 48.98)] and it specifies the date or time of events [*OR* = 22.31, *95% CI* (9.63, 57.92)]. The rumor is also likely to be true when it does not evoke positive or negative emotions [*OR* = 0.15, *95% CI* (0.08, 0.29)] and does not include a call for action [*OR* = 0.06, *95% CI* (0.02, 0.12)]. By contrast, the presence of source cues [*OR* = 0.64, *95% CI* (0.31, 1.28)] and visuals [*OR* = 1.41, *95% CI* (0.53, 3.73)] is related to this probability with limited significance. Our findings provide some clues for identifying COVID-19 rumors using their content characteristics.

## Introduction

Rumors are unverified information that circulates online and offline (1–3). Rumors regarding COVID-19 can be unverified facts, misunderstandings of facts, a pursuit of factual information, a question on current policies, or deliberate deception (2, 4–7). A recent poll in the United Kingdom showed that 46% of citizens came across rumors about COVID-19 (8). Similarly, the Pew Charitable Trusts in the United States indicated that 48% of the population had been exposed to such rumors (9).

Coronavirus disease 2019 rumors in China can be grouped into five categories: the nature of the virus, pandemic areas and confirmed cases, COVID-19 policies, authorities and organizations (e.g., WHO), and medical supplies (10). Rumors undermine the government's efforts to control the pandemic because many social media users cannot discern their authenticity (3, 11). For example, a recent British study indicated that only 4% of participants could tell fake daily news from real ones (12).

Our study explores whether the content characteristics of COVID-19 rumors in China predict the likelihood of their veracity. We have examined information length (headline and statement), information specification (place, time, source cue, and visual), and information effectiveness (emotion and call for action) as predictors of the veracity of rumors.

## Literature review

Rumors spread along with the COVID-19 pandemic and hinder the public from making informed decisions. To debunk COVID-19 rumors, many checklists, guidelines, fact-checking tools, digital training programs for health literacy, and long-term strategic plans have been implemented (11). However, these measures were not as effective as expected because they failed to help the public discern false rumors using “rules of thumb” (13–15). Moreover, although previous studies advocated improving social media users' health literacy, they did not examine the content characteristics of health rumors (16–19).

By contrast, researchers have attempted to use the content characteristics of deceptive information to discern truthful and deceptive statements (20, 21). For instance, Fuller et al. (22) demonstrated that word count was a significant indicator of deceptive information. Huang et al. (23) further revealed that deceivers were most likely to offer more information to convince receivers. Similarly, Zhou et al. (24) and Luo et al. (25) found that the false statement was longer because deceivers must provide supporting evidence and details to persuade their receivers to construe the message. In addition, some studies have examined misinformation detection using content-based features such as paralanguage features (10, 26–29). For example, Qazvinian et al. (27) reported that tweets with more hashtags were more likely to be misinformation than those without them.

Nonetheless, few studies have examined the content characteristics of rumors (29, 30). Zhou and Zhang (31) found that false rumors were more likely to have lower lexical diversity and contained more uncertain words than true rumors. Luca and Zervas (32) showed that fake reviews on Yelp tended to use more extreme words and expressions than real reviews. In addition, Zhang et al. (33) revealed that the presence of numbers was a good indicator for rumors. Chua and Banerjee (34) indicated that information using more exclusive words was more likely to be false. Furthermore, previous studies investigated whether the frequency of question marks, sentiment markers, arbitrary words, and tentative words (e.g., “maybe”) could differentiate false rumors from true ones (10, 13, 35, 36).

According to information manipulation theory (37), deceptive information misleads receivers by covertly violating the principles of quantity, quality, manner, and relevance. Thus, deceivers may create rumors by manipulating these principles. Indeed, some online rumors are generated intentionally by rumor creators who use specific writing styles with unique content characteristics to avoid being detected as rumors (9, 38). However, studies on the content characteristics of COVID-19 rumors are particularly scarce (29, 39). In addition, since previous studies are diverse in objectives, methodologies, and topics, they are insufficient for establishing a reliable and effective rumor detection system or “rules of thumb” for debunking COVID-19 rumors (35, 40).

Our study aims to address these research gaps. Following the framework proposed by Zhang and Ghorbani (29), we define the content characteristics of rumors as fundamental components of the natural language and categorize them into three types: information length, information specification, and information effectiveness. Information length refers to the number of words in the headline and the statement of rumors. Information specification refers to details of the content, including place and time of events, source cues of the information, and the use of visuals. Information effectiveness refers to emotions that rumors intend to evoke and actions that receivers are expected to take.

## Hypotheses

**Hypothesis 1 (H1):** Information with a longer headline or statement is more likely to be false.

Information manipulation theory states that deceptive information often violates the conversational principle of quantity by altering the amount of the information supplied to receivers (37). Word count is known as a significant predictor of deceptive information (22), but the directionality was not consistent in previous studies. Huang et al. (23) found that deceivers were most likely to offer more information to convince receivers. Similarly, Zhou et al. (24) and Luo et al. (25) found that deceptive information had a longer length than truthful ones since deceivers had to provide supporting evidence and details to persuade their receivers to construe this information as true. They also showed that information length was a good predictor for distinguishing true and false rumors in various topics, including politics, science, and public health. In addition, Zhang et al. (33) found that the length of both the headline and the statement was associated with the authenticity of online health rumors in China. The longer the headline and the statement, the more likely the rumor was false.

**Hypothesis 2 (H2):** Information with an ambiguous place or time of events and an ambiguous source cue is more likely to be false.

Several studies have indicated that deceptive statements contain less detailed content than truthful ones (20, 24). Deceivers may lack actual experiences and, thus, may be unable to provide detailed information. For instance, Zhang et al. (33) revealed that the presence of numbers was a good indicator of true rumors. Bond and Lee (20) found that deceptive statements were less likely to contain sensory and temporal vocabularies. Banerjee and Chua (11) found that deceptive online reviews had more diminutive nouns than authentic ones.

Moreover, online rumors often lack concrete source cues. Previous studies have focused on governmental organizations and news agencies such as the Xinhua News of China or the Cable News Network (CNN) of the USA (1, 33, 41). For example, Zhang et al. (33) examined domestic and foreign source cues and found that rumors with either type of source cues were positively related to the probability of being true. Recently, some researchers have proposed that informants in rumors can be further divided into ambiguous or concrete source cues and be used to evaluate the veracity of rumors (41–46). For instance, the source cue in the rumor, “*A doctor of Peking Union Medical College Hospital recently published an article to confirm that clearing nose with normal saline could prevent COVID-19 virus*,” refers to an ambiguous person— “a doctor.” By comparison, the source cue in the rumor “*Zhong Nanshan announced that sequela of COVID-19 was more severe than that of a severe acute respiratory syndrome (SARS)*” is concrete—Zhong Nanshan (a distinguished respiratory specialist in China).

**Hypothesis 3 (H3):** Information without visuals such as pictures or videos is more likely to be false.

Coronavirus disease 2019 rumors are often presented with visuals such as pictures and videos (14, 15, 47–51). Visuals are expected to represent reality because people tend to believe “*seeing is believing*” and “*a picture is worth a thousand words*” (52). Previous studies have found that pictures were presented with truthful information and sometimes were used to increase the perceived authenticity of rumors (14, 33, 53, 54). However, Zhang et al. (33) found that the presence of pictures did not differentiate true health rumors from false ones.

**Hypothesis 4 (H4):** Information that elicits positive or negative emotions and contains a call for action is more likely to be false.

Coronavirus disease 2019 rumors may have a long-term impact on people's emotional states by eliciting negative emotions such as anxiety, helplessness, anger, and discomfort (55–57). Nonetheless, there is no consensus on whether elicited emotions are linked with the veracity of rumors. (5) reported that sentiments predicted the veracity of rumors on Twitter. Similarly, Zhang et al. (33) found that dreadful health rumors that described fearsome, disappointing, and undesirable events or outcomes were more prevalent and were more likely to be accurate than wish rumors that described potential positive consequences. In contrast, Chua and Banerjee (34) argued that sentiments elicited by rumors could not predict the veracity.

Furthermore, providing false information is not the only negative impact of false rumors. Many COVID-19 rumors may end with a call for action, such as transmitting the message to friends or relatives (39). This call for action may also motivate receivers to act against COVID-19 policies, cause panic among the public, and urge receivers not to take vaccines (13, 21, 45, 58–60). However, whether a call for action is linked to the veracity of rumors is not yet known.

## Method

In this study, we collected rumors listed on piyao.org.cn (hereafter known as *piyao*), an official rumor-debunking platform run by the Xinhua News of China and the Cyberspace Administration of China. This website collects rumors from 31 other major rumor-debunking platforms in China, including Zhuoyaoji (捉妖记), Wenzhoupiyao (温州辟谣), and real-time refutation of COVID-19 rumors (新冠实时辟谣). According to *the 47th China Statistical Report on Internet Development* (61), *piyao* releases and refutes most rumors spreading in China and attracts 100 million visits yearly. Like Snopes.com, *piyao* allows online users to submit rumors. With the help of professionals, researchers, and reporters, *piyao* sets the record straight on every rumor that it collects by rating it as “true,” “false,” or “undetermined” (33, 62). In addition, facing the COVID-19 pandemic, *piyao* added a section dedicated to “COVID-19 rumor-debunking” and has become an authoritative platform for debunking COVID-19 rumors. A rumor on *piyao* is presented in its original form (including a headline and a statement, and sometimes visuals if it contains) along with a veracity rating, checked facts, and detailed analyses of the information.

In total, 1,685 COVID-19 rumors were collected from *piyao* in January 2022. All the rumors had headlines and statements. We assume that veracity ratings from *piyao* represent the truth. Three hundred eighty-nine rumors were categorized as “undetermined” and, thus, excluded, leaving 1,296 rumors in the final data analysis. Table 1 presents the examples of rumors and their content characteristics. Table 2 shows the coding schemes. Two coders (JZ and CF) undertook the coding in two phases. In the first phase, they worked independently on 168 randomly-selected rumors and then resolved their disagreements (if any) through in-person discussions. The two coders demonstrated almost perfect inter-rater reliability for all the measures as indicated by Cohen's kappa (k) (place: *k* = 0.95, *p* < 0.001; time: *k* = 0.98, *p* < 0.001; source cue: *k* = 0.95, *p* < 0.001; visual: *k* = 1.00, *p* < 0.001; emotion: *k* = 0.95, *p* < 0.001; call for action: *k* = 0.96, *p* < 0.001). In the second phase, they coded the remaining rumors that were assigned to them randomly.

**Table 1:** Examples of COVID-19 rumors and their content characteristics.

**Content characteristics**		**Description**	**Example**
Information length	Headline	The number of Chinese characters in a rumor headline	Attention! Attention! A technique to get! Medical doctors confirmed that normal saline to prevent COVID-19. (注意！注意！教大家一个新技能！医生证实生理盐水可以预防新冠病毒。)
	Statement	The number of Chinese characters in a rumor statement	Surprise! A Peking Union Medical College Hospital doctor recently published a paper confirming that clearing the nose with normal saline could prevent the COVID-19 virus. To protect your family, please retransmit this information to your relatives and friends with your fingers. (惊喜！北京协和医院的一个医生近日发文，可以用生理盐水清洗鼻子，这样能有效预防新型冠状病毒感染。为了让你的家人远离新冠病毒，动动你的手指，转发给你的家人和朋友。)
Information specification	Place	Concrete or ambiguous place	Peking Union Medical College Hospital (北京协和医院)
	Time	Concrete or ambiguous time	recently (近日)
	Source cue	Concrete or ambiguous source cues	a doctor (一个医生)
	Visual	Presence of pictures or videos along with the text	pictures/videos
Information affectiveness	Emotion	A positive or negative emotion that expresses hope, happiness, relief, disgust, anger, fear, and anxiety	protect your family (保护家人)
	Call for action	A call for action such as sending this message to friends	Please retransmit this information to your relatives and friends (动动你的手指，转发给你的家人和朋友)

**Table 2:** Coding schemes for the content characteristics of COVID-19 rumors.

**Variable**	**Coding scheme**
Veracity	True rumors = 1, False rumors = 0
Headline	Number of Chinese characters in the headline
Statement	Number of Chinese characters in the statement
Place	Concrete place = 1, Ambiguous place = 0
Time	Concrete time = 1, Ambiguous time = 0
Source cue	Concrete source cue = 1, Ambiguous source cue = 0
Visual	With visual = 1, Without visual = 0
Emotion	With emotion =1, Without emotion = 0
Call for action	With a call for action=1, Without a call for action = 0

## Results

Tables 3–5 present the descriptive statistics of our data. In total, there were 82% false rumors and 18% true rumors out of 1,296 rumors in our sample. The Shapiro–Wilk test showed that the headline (*W* = 0.95, *p* < 0.001) and the statement (*W* = 0.70, *p* < 0.001) are not normally distributed. We first conducted preliminary analyses to examine the associations between the veracity of rumors and each independent variable. For the headline and the statement, we ran the Wilcoxon rank-sum test to examine whether true and false rumors showed significant differences in the number of Chinese characters. We found that the headline of the false rumor contained significantly more Chinese characters (*Mdn* = 12) than the true rumor (*Mdn* = 8, *p* < 0.001, *r* = −0.42). The statements of the false rumor (*Mdn* = 78) contained significantly fewer Chinese characters than the true rumor (*Mdn* = 120, *p* < 0.001, *r* = −0.53). In addition, separate Pearson's chi-squared test showed that there were significant associations between the veracity of rumors and whether the information contained a concrete place (*X*^2^ = 37.85, *p* < 0.001) with an odds ratio of 2.45, a concrete time (*X*^2^ = 48.44, *p* < 0.001) with an odds ratio of 2.74, a concrete source cue (*X*^2^ = 39.06, *p* < 0.001) with an odds ratio of 2.46, a visual (*X*^2^ = 12.65, *p* < 0.001) with an odds ratio of 0.51, an emotion (*X*^2^ = 148.24, *p* < 0.001) with an odds ratio of 0.17, and a call for action (*X*^2^ = 140.17, *p* < 0.001) with an odds ratio of 0.17.

**Table 3:** Descriptive statistics of information length.

	**Full dataset (*****n*** = **1,296)**			**True rumors (*****n*** = **232)**			**False rumors (*****n*** = **1,064)**		
**Variable**	**Range**	**Mean**	**Median**	**Range**	**Mean**	**Median**	**Range**	**Mean**	**Median**
Headline	[5, 28]	13.16	12	[5, 17]	8.73	8	[6, 28]	14.12	12
Statement	[17, 330]	92.38	80	[78, 330]	143.03	120	[17, 201]	81.37	78

**Table 4:** Descriptive statistics of information specification.

	**Full dataset (*****n*** = **1,296)**		**True rumors (*****n*** = **232)**		**False rumors (*****n*** = **1,064)**	
**Variable**	**%**	**%**	**%**	**%**	**%**	**%**
	**(Value=1)**	**(Value=0)**	**(Value=1)**	**(Value=0)**	**(Value=1)**	**(Value=0)**
Place	38.67 (501)	61.33 (795)	46.65 (108)	53.35 (124)	36.88 (392)	63.12 (672)
Time	42.32 (548)	57.68 (748)	63.31 (147)	36.69 (85)	37.77 (402)	62.23 (662)
Source cue	35.71 (463)	64.29 (833)	56.68 (132)	43.32 (100)	31.24 (333)	68.76 (731)
Visual	31.04 (402)	68.96 (894)	16.72 (39)	83.28 (193)	34.16 (364)	65.84 (700)

**Table 5:** Descriptive statistics of information affectiveness.

	**Full dataset (*****n*** = **1,296)**		**True rumors (*****n*** = **232)**		**False rumors (*****n*** = **1,064)**	
**Variable**	**%**	**%**	**%**	**%**	**%**	**%**
	**(Value=1)**	**(Value=0)**	**(Value=1)**	**(Value=0)**	**(Value=1)**	**(Value=0)**
Emotion	59.88 (776)	40.12 (520)	24.73 (57)	75.27 (175)	67.01 (713)	32.99 (351)
Call for action	58.95 (764)	41.05 (532)	26.74 (62)	73.26 (170)	65.93 (701)	34.07 (363)

However, the above preliminary analyses could not include all the independent variables simultaneously. We, thus, further used logistic regression to examine the relationship between the veracity of rumors and eight independent variables in a single model. Our analysis met all the assumptions of using the logistic regression, including: (1) the response variable (veracity) is binary, (2) the observations are independent, (3) there is no multicollinearity among explanatory variables as indicated by low variance inflation factor (VIF) values (headline: 5.00; statement: 3.88; place: 1.82; time: 2.16; source cue: 1.34; visual: 1.16; emotion:1.08; and call for action: 1.82), (4) there are no extreme outliers, and (5) the sample size was determined based on the number of independent variables. We found that a minimum sample size of 500 yields reliable and valid sample estimates (63). Thus, our sample size is sufficient.

Table 6 shows the findings of the logistic regression with all the eight independent variables as predictors for the veracity of COVID-19 rumors in China. The statistical significance of each predictor was corrected using the false discovery rate (FDR) with the Benjamini–Hochberg method. We found that the number of Chinese characters in a rumor's headline (*p* < 0.001) and statement (*p* < 0.001) were both significantly related to the veracity, supporting H1. The presence of a concrete place (*p* < 0.001) or time of events (*p* < 0.001) was also significantly related to the veracity of rumors, supporting H2. Nonetheless, source cues (*p* = 0.214) and visual (*p* = 0.492) were associated with the veracity with limited significance, thus not supporting H3. Furthermore, we found that a call for action (*p* < 0.001) was significantly related to the veracity, supporting H4.

**Table 6:** Logistic regression findings.

**Predictors**	**Odds Ratios**	**95% CI**	**p**	**FDR corrected p**
Headline	0.37	0.30–0.44	<0.001	<0.001
Statement	1.11	1.09–1.13	<0.001	<0.001
Place	20.83	9.60–48.98	<0.001	<0.001
Time	22.31	9.63–57.92	<0.001	<0.001
Source	0.64	0.31–1.17	0.214	0.244
Call	0.06	0.02–0.12	<0.001	<0.001
Visual	1.41	0.53–3.73	0.492	0.492
Emotion	0.15	0.08–0.29	<0.001	<0.001

*N = 1,296, R^2^ = 0.78*.

## Discussion

The Internet is a vital and convenient platform for spreading information about the COVID-19 pandemic (7, 33, 64). However, there is also a sharp increase in COVID-19 rumors. The ratio of true rumors varies across studies in different contexts and topics. Our data showed that false COVID-19 rumors (82%) were much more prevalent than true rumors (18%) in China. This finding is consistent with Zhang et al. (33), who revealed that 75.1% of 453 health-related information was false. It contradicted Gelfert (65) that claimed rumors were often based on facts and were, thus, usually true.

Importantly, our findings support most of our hypotheses. First, although the length of the headline and statement are significant indicators of the veracity of rumors, we found opposite effects. The longer the headline, the more likely the rumor was false. By contrast, the shorter the statement, the more likely the rumor was false. Our finding about the length of the headline is consistent with prior studies (25, 66–69). However, our finding regarding the length of the statement contradicts previous studies (70–72), which revealed that deceptive statements were longer than truthful ones because deceivers attempt to provide more information to increase the perceived credibility of the information. Similarly, Zhang et al. (33) showed that false health-related rumors were longer than true ones because rumormongers have learned that longer statements could reduce uncertainty.

We speculate that there are two possible explanations for these contradictory findings. One possibility is that rumormongers have learned to take advantage of their target readers' dependence on the Internet and smartphones for information acquisition. The long headline is, thus, used to increase the attractiveness and saliency of the information to catch readers' attention. However, as the screen size of smartphones is limited, rumormongers may tend to avoid lengthy details so readers can finish reading the complete statement instead of a piece of fragmented information on one page. The other possibility is that since rumormongers have not witnessed the events, it is difficult for them to provide detailed information. Previous studies have shown that rumormongers could only add peripheral information, but not key details (73–75).

Second, we found that the presence of concrete place and time of events are good indicators of true rumors. Prior studies have revealed that deceptive information included vague spatial and temporal information (10, 39), while true information contained more details. Our findings are consistent with these studies. However, our results differed from Zhang et al. (33) that revealed the presence of a place name was related to the veracity of a rumor with limited significance. However, one crucial difference between Zhang et al. (33) and our study is that we categorized the “place” into two types: an ambiguous place and a concrete place. We found that only when a concrete place is included in a rumor, it is more likely to be true.

Similarly, we found that rumors containing a concrete time of events such as “on Wednesday” or “in October” rather than an ambiguous time such as “these days” or “recently” are more likely to be true. These findings are consistent with previous studies on deception, which have demonstrated rumormongers may feel strain, guilt, and restlessness in the process (20–22). So, they tended to provide ambiguous information to maintain their distance from the false information receivers (76, 77). Moreover, rumormongers may give such details in a fuzzy, nebulous manner (11, 76) because information receivers could easily use a concrete place and time of events to verify and debunk false rumors.

Third, we did not find any significant association between source cues and the veracity of rumors, which contradicts previous studies (9, 10, 71). Nonetheless, our finding is consistent with other studies that have been conducted in China. For example, Zhou et al. (24) and Jang et al. (39) revealed that rumormongers counterfeited celebrities' sayings with expressions such as “Professor Zhong Nanshan warned that…” or “Professor Zhang Wenhong said…” Despite containing specific source cues, these rumors were often false.

Fourth, we did not find any significant association between the use of visuals and the veracity of rumors. Previous studies argued that rumormongers used visuals to strengthen the perceived credibility of information. For example, Zhang et al. (33) revealed that the use of pictures was negatively related to the veracity of rumors. Nonetheless, visuals may not be related to the veracity of the rumors because rumormongers often included pictures or videos that were not associated with the events (78–80) or used fake pictures or videos (48, 76, 81) as evidence to imitate the practice of news reports.

Fifth, we showed that elicited emotions in rumors could differentiate between true and false rumors. This finding is inconsistent with Zhang et al. (33), which found that health-related rumors evoking positive emotions were more likely to be false than negative ones. Nonetheless, our finding is consistent with Li et al. (82), which demonstrated that heightened emotions were associated with the veracity of the information. Both the positive and negative emotions increase uncritical acceptance of information. These discrepancies may lie in the varied backgrounds of these studies. Our study focuses on COVID-19 rumors that often involve high emotional effectiveness, but Zhang et al. (33) focused on health-related rumors that may not contain a high ratio of emotions.

Finally, we found that the presence of a call for action is associated with false rumors. Previous studies have also shown that calling for counteraction to create chaos could be the purpose of some rumors (83, 84). Whether a rumor contains a call to retweet the information or a call to take counteraction against governmental prevention and curative policies may present an obstacle to implementing these policies.

## Implications and limitations

This study has several theoretical contributions. First, our findings show that information manipulation theory (37) may not apply to all the topics. Deceivers utilize different information manipulation techniques in various topics or fields. For instance, deceivers often create longer statements to enhance perceived credibility, while online COVID-19 rumors usually have shorter statements. Second, this study sheds light on the research field of “using online data to predict human behaviors” by offering a tentative linguistic approach. Our findings show that the content characteristics of rumors predict the veracity of rumors, which may facilitate information receivers to differentiate between false and true rumors. Third, this study extends our current knowledge of information identification by exploring the content characteristics of COVID-19 rumors. A few rules of thumb should be followed when evaluating the veracity ratings of online COVID-19 rumors, with particular attention to the length of headlines and statements, concrete place and time of events, and the presence of emotion and a call for action.

From a practical perspective, the findings of this study can be used as “rules of thumb” to fight against false COVID-19 rumors. Health agencies, organizations, and institutions may use better countermeasures to fight against false-rated rumors. For instance, they may analyze and summarize the content characteristics of each rumor after presenting factual information. This practice may enhance people's skills in identifying false rumors. In addition, our findings may be used as references for evaluating factual information and for debunking putatively false rumors. We suggest that the content characteristics such as place and time of events should be carefully examined. Furthermore, our study focused exclusively on the content characteristics of COVID-19 rumors in contrast to most prior studies. Facing the prevalence of COVID-19 rumors online, our study offers a set of easy-to-follow guidelines for evaluating the veracity of these rumors by online users.

Our study has some limitations. First, our study only focuses on COVID-19 rumors in China. Our findings may not generalize to COVID-19 rumors spreading in other languages or other countries. Future studies may examine the content characteristics of COVID-19 rumors in different societies and cultures. Another limitation is that our study does not provide any causal links between the content characteristics and the veracity of rumors. In addition, as our study only examines a limited number of the content characteristics of COVID-19 rumors, further studies may explore more features that might be useful in distinguishing true and false rumors using text mining techniques.

## Conclusion

Our study provides preliminary evidence on the use of the content characteristics of rumors as guidelines to fight against false COVID-19 rumors. We found that information receivers should pay particular attention to the place and time of events and evaluate whether these rumors include concrete or an ambiguous place and time of events. We also revealed that information receivers should check the length of the headline and the statement, assess whether the information elicits any emotion, and watch out for a call for action. Nonetheless, the presence of source cues or visuals may not help differentiate between true and false rumors.

## Data availability statement

The raw data supporting the conclusions of this article will be made available by the authors, without undue reservation.

## Author contributions

CF designed the study. JZ performed the research. CF and JZ analyzed the data. CF, JZ, and XK wrote the paper. All authors declare no competing interest and agree the final version of this manuscript.

## Funding

This study was funded by the National Social Science Fund of China (18BYY066), the Fundamental Research Funds for the Central Universities of Chongqing University (2021CDJSKZX10 and 2022CDJSKJC02), and Grants in Humanities and Social Sciences by Chongqing Municipal Education Commission (22SKJD006).

## Conflict of interest

The authors declare that the research was conducted in the absence of any commercial or financial relationships that could be construed as a potential conflict of interest.

## Publisher's note

All claims expressed in this article are solely those of the authors and do not necessarily represent those of their affiliated organizations, or those of the publisher, the editors and the reviewers. Any product that may be evaluated in this article, or claim that may be made by its manufacturer, is not guaranteed or endorsed by the publisher.
